# Two New Cave-Dwelling Species of the Short-Tailed Whipscorpion Genus *Rowlandius* (Arachnida: Schizomida: Hubbardiidae) from Northeastern Brazil, with Comments on Male Dimorphism

**DOI:** 10.1371/journal.pone.0063616

**Published:** 2013-05-22

**Authors:** Adalberto J. Santos, Rodrigo Lopes Ferreira, Bruno A. Buzatto

**Affiliations:** 1 Departamento de Zoologia, Instituto de Ciências Biológicas, Universidade Federal de Minas Gerais, Belo Horizonte, Minas Gerais, Brazil; 2 Departamento de Biologia, Universidade Federal de Lavras, Lavras, Minas Gerais, Brazil; 3 Centre for Evolutionary Biology, School of Animal Biology (M092), The University of Western Australia, Perth, Western Australia, Australia; Australian Museum, Australia

## Abstract

Two new species of the arachnid order Schizomida, *Rowlandius ubajara* **sp.nov.** and *Rowlandius potiguar* **sp.nov.**, are described based on both male and female specimens collected in caves from northeastern Brazil. *Rowlandius ubajara* is known only from the Ubajara Cave, in the state of Ceará; *R. potiguar* is recorded from 20 caves of the Apodi Limestone Group, in the state of Rio Grande do Norte. A remarkable dimorphism in male pedipalp length is described and analyzed in *R. potiguar*. The distribution of male pedipalp length is clearly bimodal in the species, but the two male morphs (homeomorphic and heteromorphic) present some overlap in the sizes of this structure. Moreover, males show a steeper allometry in pedipalp length than females, indicating that this trait is under a different selective regime in males and in females.

## Introduction

The arachnid order Schizomida, the short-tailed whipscorpions, is represented in South America mainly in northern forested areas. Besides its great diversity and broad distribution in Central America and the West Indies, in the continental South America it can be considered a poorly-known Amazonian group. Until very recently, the only species known from southern parts of the subcontinent was *Stenochrus portoricensis* Chamberlin, 1922 [1], [2], a widespread and synanthropic species. However, the scarcity of schizomid species outside the Amazonian forest can be attributed, at least in part, to collecting bias. This happens because schizomids occur mostly in poorly sampled habitats, like the soil leaf litter, termite and ant nests, and caves [2], [3]. Subterranean habitats in particular can harbor large schizomid populations [3], and are a promising source of undescribed species.

Among South American countries, Brazil is known for its abundance of caves, which are scattered in several geological formations [4]. Despite the richness of cave habitats, the Brazilian subterranean fauna is mostly unknown [5], though it is currently attracting the interest of many biologists.

The genus *Rowlandius* Reddell and Cokendolpher, 1995, the focus of this study, is a good example of how little is known about the schizomids and the cave fauna of Brazil. The majority of the 54 species of the genus [2], [6], [7], [8], [9], [10] occur in Caribbean islands, mainly Cuba, Jamaica and Hispaniola (see for instance [11], [12], [13], [14], [15]). The genus is represented in continental South America by one species in Venezuela (*R. arduus* Armas, Villareal & Colmenares, 2009), one in the eastern Brazilian Amazonia (*R. sul* Cokendolpher & Reddell, 2000) and another, recently described species from the northeastern Brazilian Atlantic Forest (*R. linsduarteae* Santos, Dias, Brescovit & Santos, 2008) [8]. In this study, two new species are described based on specimens collected in cave habitats in northeastern Brazil. Most caves from which specimens were retrieved are imbedded in the caatinga, a northeastern Brazilian, semi-arid biome [16], an unexpected habitat for a group mostly considered as a humid forest dweller.

Several species of *Rowlandius* and other genera of schizomids are known by the remarkable variation in the length of male pedipalp. Depending on the species, the length of the male pedipalp can vary from approximately the same size as those of the female to up to three times as long (e.g. [11], [15]). Authors describing species of these genera usually discriminate males in two categories, namely homeomorphic males and heteromorphic males, for specimens with short and long pedipalps, respectively [11]. This classification is based on the assumption that two discrete male morphs can be recognized based on the pedipalp size, relative to total body size. Male dimorphism has been described for several groups of arthropods, like insects [17], crustaceans [18] and, among arachnids, spiders [19], mites [20], pseudoscorpions [21] and harvestmen [22], [23]. This phenomenon has been attributed to intrasexual selection pressures favoring a conditional strategy with different tactics for small and large males [24]. However, the detection of male dimorphism can be statistically challenging, especially when the differences between the morphs are subtle [25]. For schizomids, no criteria have been proposed to discriminate between homeomorphic and heteromorphic males, and this distinction is generally based on small sample sizes, usually no more than five or six specimens. In this study, we take advantage of a large sample of specimens to evaluate whether or not it is possible to recognize discrete male morphs in one of the newly described species.

## Materials and Methods

### Specimens and Laboratory Procedures

**Figure 1:**
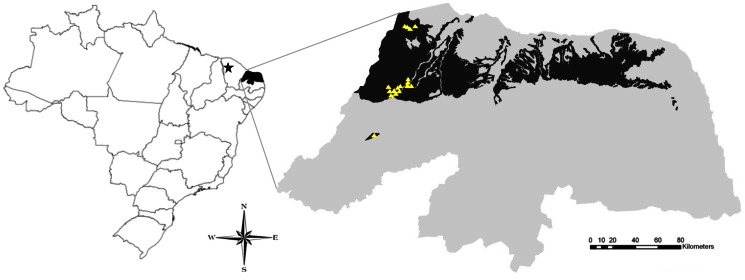
Geographic distribution records of *Rowlandius ubajara* sp.nov. (star) and *R. potiguar* sp.nov. (triangles) in northeastern Brazil. The black area represents the range of limestone outcrops from the Apodi Group in the state of Rio Grande do Norte.

The specimens examined for this study were collected in several caves in two states in northeastern Brazil (Fig. 1) and were deposited in the arachnid collections of Coleção de Invertebrados Subterrâneos de Lavras, Universidade Federal de Lavras, Lavras (ISLA, curator R.L. Ferreira); Coleções Taxonômicas da UFMG, Belo Horizonte (UFMG, A.J. Santos); Departamento de Zoologia, Universidade de Brasília, Brasília (DZUB, P.C. Motta) and Instituto Butantan, São Paulo (IBSP, D.M. Barros-Battesti). Field collecting permits were issued to R.L. Ferreira by SISBIO/CECAV (license n. 14783-1). Cave geographic coordinates were obtained through CECAV’s database (available at http://www4.icmbio.gov.br/cecav/index.php?id_menu=228). Specimens were illustrated in a Leica MZ12.5 stereomicroscope with a camera lucida and measured in an Olympus SZ40. Female genitalia were excised, temporarily mounted in slides with clove oil and illustrated in a Zeiss Axioskop 20 binocular microscope with a camera lucida. Specimens for SEM were air-dried, mounted on stubs with double-faced tape and sputter-coated with 10 nm of gold. Stubs were examined and photographed under high vacuum in a Quanta 2000 Scanning Electron Microscope at the Centro de Microscopia da UFMG. Female genitalia was excised, immersed for two hours in a pancreatin solution (see [26]) to remove soft tissues, air-dried and mounted on the stub. Description format and abbreviations follow Santos *et al*. [2] and Santos & Pinto-da-Rocha [27]. Morphological nomenclature follows [28] for chelicerae setation, [29] and [30] for flagellum setation and [3] for female internal genitalia. All measurements are in millimeters.

### Male Dimorphism Analysis

Male dimorphism was at first visually evaluated in scatterplots of prosoma length (as a proxy for general body size) and pedipalpal patella length (indicative of pedipalp size). To check whether a non-linear or discontinuous allometric pattern was apparent, natural values were used without logarithm transformation (caveats of such transformations explained in [31]), and the scatterplot axes were isometric (such that ΔX = ΔY; see [32] for why this is important). Both variables were checked for bimodality through adjustment of non-parametric kernel density estimates to frequency distributions. Pedipalp size distribution seemed strongly bimodal, and therefore was parameterized as a mixture of two ‘facing gamma distributions’ using finite mixture models, according to model <d> in [33]. The ‘facing gamma distributions’ proposed by these authors accommodate skews in the distributions of the trait for each male morph, modeling a skew to the right (towards higher values) among homeomorphic males and a skew to the left (towards lower values) among heteromorphic males. This parameterization was performed in SAS version 9.2 (SAS Institute 2004) with code kindly provided by J.M. Rowland and C.R. Qualls. Finally, the likelihood of belonging to each male morph was computed from the mixture models for each male based on their pedipalp length.

Simple linear models were fitted to the allometric relationship between pedipalp length and prosoma length for females and for each male morph separately, through standard major axis regression using package ‘lmodel2’ [34] in R version 2.14.2 [35]. For this last step of the analysis, males were classified as homeomorphic or heteromorphic when their likelihood of belonging to one particular male morph (computed from the mixture models) was higher than 95%. Males with less than 95% chances of belonging to either male morph were left out when adjusting the allometric relationships between pedipalp length and prosoma length for homeomorphic and heteromorphic males.

### Nomenclatural Acts

The electronic edition of this article conforms to the requirements of the amended International Code of Zoological Nomenclature [36], and hence the new names contained herein are available under that Code from the electronic edition of this article. This published work and the nomenclatural acts it contains have been registered in ZooBank, the online registration system for the ICZN. The ZooBank LSIDs (Life Science Identifiers) can be resolved and the associated information viewed through any standard web browser by appending the LSID to the prefix "http://zoobank.org/". The LSID for this publication is: (urn:lsid:zoobank.org:pub:4882594A-5176-4668-A8B3-20DFFDD4DA73). The electronic edition of this work was published in a journal with an ISSN, and has been archived and is available from the following digital repositories: PubMed Central, LOCKSS.

## Results and Discussion

### Taxonomic Treatment

#### Rowlandius ubajara sp.nov

**Figure 2:**
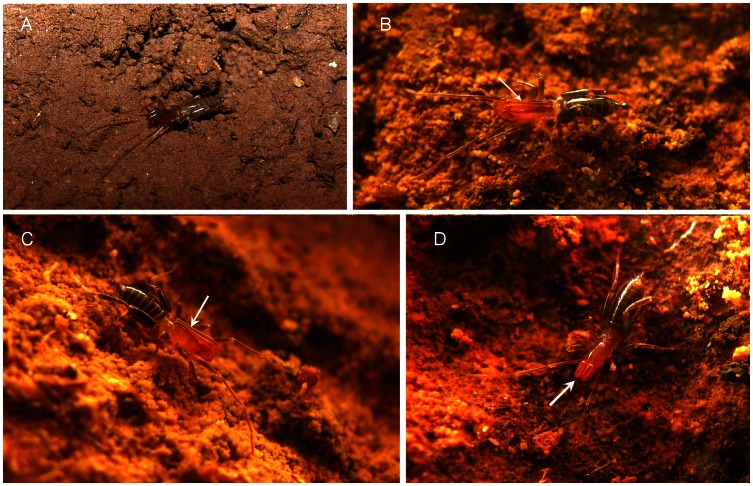
New species of *Rowlandius* from northeastern Brazil. (A) *Rowlandius ubajara* **sp.nov.**, female from Ubajara, Ceará, Brazil. (B, C) *Rowlandius potiguar* **sp.nov.**, male from Felipe Guerra, Rio Grande do Norte, Brazil. Note the extremely elongated pedipalp (arrow). (D) *R. potiguar*, female from Felipe Guerra, Rio Grande do Norte, Brazil (arrow indicates the pedipalp).

**Figure 3:**
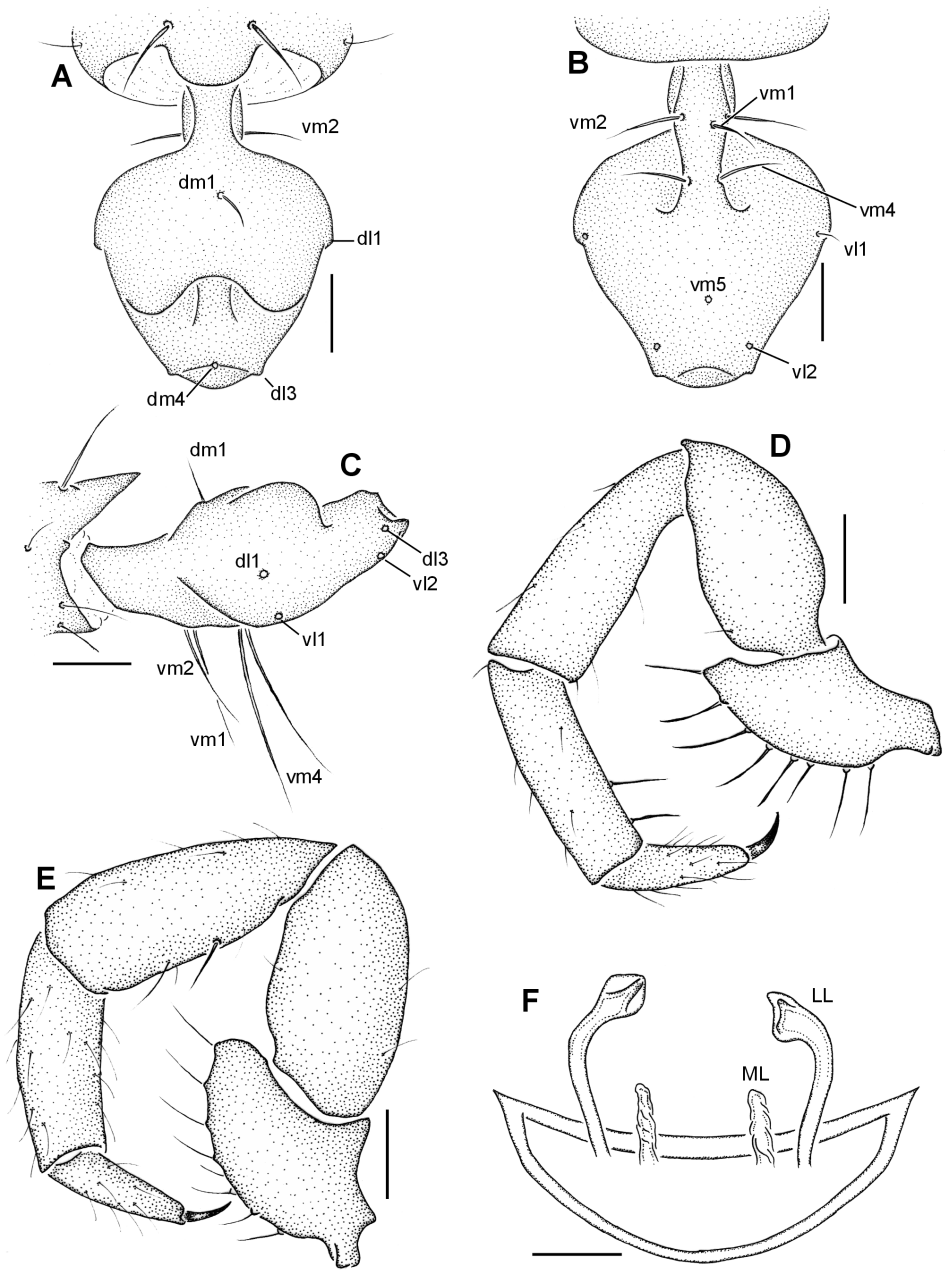
*Rowlandius ubajara* sp.nov. (A) Male (holotype UFMG 3895), flagellum, dorsal view; (B) ventral, (C) lateral, (D) pedipalp, retrolateral view. (E) Female (paratype, IBSP 44), pedipalp, retrolateral view; (F) female internal genitalia, dorsal view (LL lateral lobes of the spermathecae, ML median lobes). Scale bars: 6–8 0.1 mm; 9–10 0.2 mm; 11 0.04 mm.

urn:lsid:zoobank.org:act:FD09629C-2619-433C-BB0E-92B628D8596C.

(Figures 1, 2A, 3A–F).

#### Type material

Holotype. Male from BRAZIL: *Ceará*: Ubajara (Parque Nacional de Ubajara, Gruta de Ubajara, 3^o^50′0′′S 40^o^54′1′′W), 30.XII.2006, R.L. Ferreira coll. (UFMG 3895). Paratypes. Female, same data as the holotype (UFMG 3896); male and female, same data (IBSP 44); female, same locality, 26.IV.2003, B.C. Cabral coll. (DZUB 2718).

### Etymology

The specific epithet is a noun in apposition from the type locality.

### Diagnosis

Males of *R. ubajara* can be distinguished from the remaining species of the genus by the flagellum ovoid, wider at the basal third, with the anterior surface between setae dl3 wide and convex and with the dorsal projections wide, separated by a distance equivalent to their width and not touching or surpassing the lateral border of the flagellum (Fig. 3A, B). Females are recognized by the chitinized arch of the internal genitalia u-shaped, closed and pointed laterally, lateral lobes of the spermathecae with curved stalks and small bulbs and the median lobes cylindrical, wrinkled and with half the length of the lateral lobes (Fig. 3F).

### Description

Male (holotype). Propeltidium pale brown, chelicerae and pedipalp reddish-brown. Metapeldium and legs pale-brown. Opisthosoma greenish-brown. Anterior process with one apical seta. Propeltidium with an anterior and a posterior pair of setae. Eyespot absent. Opisthosomal tergites I–IX each with a dorsal pair of setae, X–XI with a pair of lateral setae and a transversal row of five setae, XII with a dorsal and two lateral pairs and a ventral row of four setae. Segments XI–XII telescoped. Posterodorsal abdominal process wide, subrectangular (Fig. 3A). Fixed digit of chelicerae with four teeth, the ventral the longest. Chelicerae setation: 1: 3; 2: 5; 3: 3; 4: 2; 5: 6; 6: 1. Setae 1 with spicules, setae 3 pilose in the apex. Movable digit with serrula and a row of pilose bristles, guard tooth inconspicuous. Pedipalp with the trochanter slightly pointed anteriorly (Fig. 3D). Leg tarsal segments proportion 2:1:2. Femur IV robust, anterior margin produced dorsally, approximately two times longer than wide. Total length 2.72. Propeltidium 1.02 long, 0.56 wide. Prosoma 1.27 long, opisthosoma 1.7. Length of pedipalp segments: trochanter 0.2/femur 0.43/patella 0.48/tibia 0.43/tarsus 0.23/total 1.77. Length of leg segments: **I** trochanter 0.18/femur 1.12/patella 1.45/tibia 1.09/basitarsus 0.31/telotarsus 0.56/total 4.71. **II** 0.08/0.71/0.41/0.43/0.41/0.36/2.4. **III** 0.1/0,58/0.31/0.33/0.15/0.28/1.75. **IV** 0.13/1.03/1.12/0.64/0.18/0.23/3.33 Flagellum ovoid, with stalk short and wide (Fig. 3A–C). Setation of flagellum: dm1, dm4; dl1, dl3; vm1, vm2, vm4, vm5; vl1, vl2 (Fig. 3A–C); five lateral microsetae between dl1 and dl3.

Female (Paratype, UFMG 3896). As in male, except by the following. Prosoma, pedipalp and leg I reddish-brown (Fig. 2). Opisthosoma, chelicerae and legs II–IV greenish brown (Fig. 2). Anterior process with 1+1 setae. Flagellum with four segments, setation as in the male, except by three pairs of lateral microsetae: one pair in segment II (adjacent to dm1); one in III and one in IV (between dm4 and dl3). Fixed finger of chelicerae with five teeth, the ventral the longest and the dorsal rounded. Movable digit with conspicuous guard tooth. Total length 4.12. Propeltidium 1.23 long, 0.66 wide. Prosoma 1.7 long, opisthosoma 2.42. Length of pedipalp segments: trochanter 0.22/femur 0.58/patella 0.61/tibia 0.53/tarsus 0.28/total 2.22. Length of leg segments: **I** trochanter 0.18/femur 1.25/patella 1.55/tibia 1.14/basitarsus 0.34/telotarsus 0.53/total 4.99. **II** 0.08/0.86/0.48/0.53/0.46/0.36/2.77. **III** 0.13/0.74/0.31/0.41/0.51/0.41/2.51. **IV** 0.28/1.19/0.53/0.83/0.76/0.41/4.00. Spermathecae without gland duct openings (Fig. 3F).

### Variation

Length of prosoma: male (N = 2) 1.27–1.37, female (N = 3) 1.26–1.73. Length of pedipalpal patella: male 0.48–0.6, female 0.53–0.66.

### Natural History

This species was recorded from a single limestone cave located in a small patch of Brazilian Atlantic forest in northeastern Brazil (in Ubajara National Park). Part of the cave is open for visitors and is illuminated by electric lamps. Specimens of *R. ubajara* were found in the inner part of the cave, which consists of a single passage through which a small stream flows. Potential food resources observed in the cave are mainly bat guano from insectivorous and hematophagous bats and potential prey for *R. ubajara* could be mainly springtails (Collembola) and booklice (Psocoptera: Psyllipsocidae). The population is apparently small, and only about 10 specimens were observed in the cave during the collection. The average temperature of the cave was 23.5°C and the humidity was 99% in the inner parts, where the species occurs. Although the population only occurs in the inner part of the cave and the species was observed in a single cave, the absence of eye spots is the only potentially troglomorphic trait exhibited by the species. This characteristic, as well as the absence of pigmentation and elongation of appendages, is frequently suggested as evidence of troglobiotism in schizomid species [3], [37]. However, it is not uncommon to find schizomids with those traits associated with epigean habitats, as well as species without any apparent troglomorphism living in caves ([37] and references therein). It is reasonable to consider schizomids as pre-adapted for living in caves, mostly due to their low reliance on visual orientation. Except for four species with convex eye lenses [38], [39], the members of the order have either no eyes or only vestigial, pale eye spots [3]. It is uncertain whether the eye spots, or even the well formed eyes, are truly functional, since no species have been evaluated through histological or behavioral methods. Thus, the presence or degree of development of the eyes does not seem to be a good indicator of adaptation to caves in this group. Since not all troglobiotic species necessarily possess troglomorphic traits [40], further studies should be conducted in the area (especially in the external environments) to actually confirm the degree of association of this species with hypogean habitats.

### Distribution

Known only from the type locality in the northeastern Brazilian state of Ceará (Fig. 1).

#### Rowlandius potiguar sp.nov

**Figure 4:**
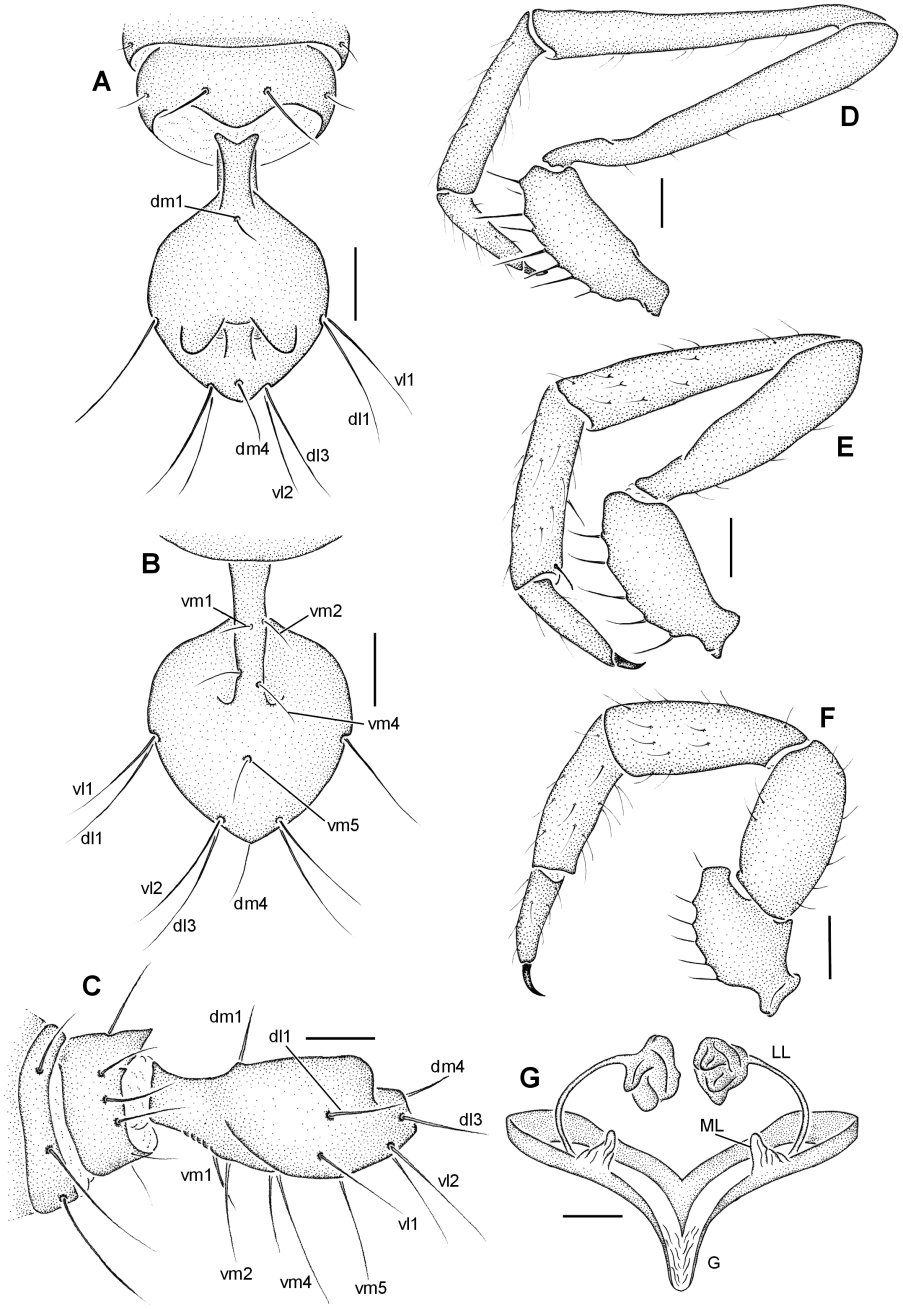
*Rowlandius potiguar* sp.nov. (A) Male (holotype, UFMG 3897), flagellum, dorsal view; (B) ventral, (C) lateral, (D) pedipalp, retrolateral view. (E) Male (paratype UFMG 3899), pedipalp, retrolateral view. (F) Female (paratype, IBSP 45), pedipalp, retrolateral view; (G) female internal genitalia, dorsal view (G gonopod, LL lateral lobes of the spermathecae, ML median lobes). Scale bars: 12–14 0.1 mm; 15–17 0.2 mm; 18 0.04 mm.

**Figure 5:**
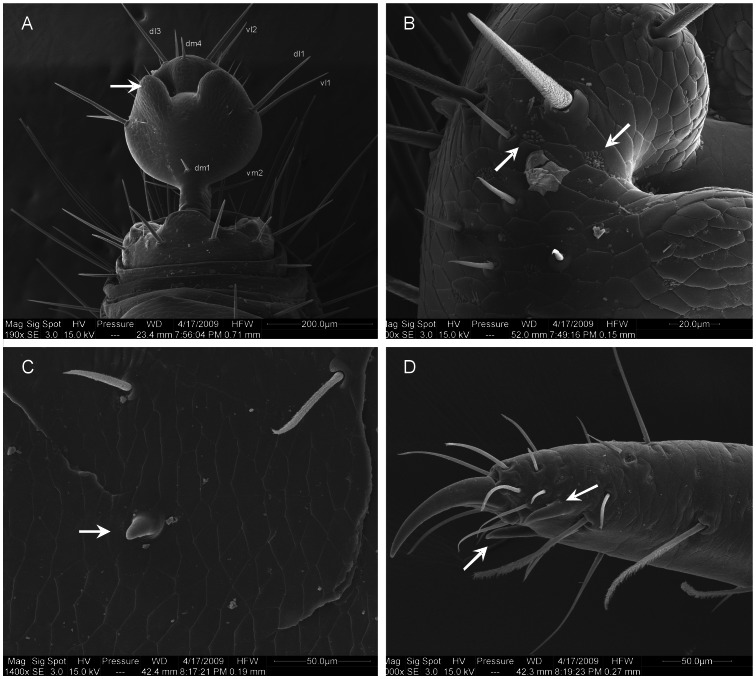
*Rowlandius potiguar* sp.nov. (A) Male (UFMG 3901), flagellum and posterior tip of opisthosoma, posterior-dorsal view, (B) apex of male flagellum, lateral view (arrows indicate uropygid pores), (C) male pedipalp, trochanter, prolateral view (arrow indicates prolateral spur), (D) male pedipalp, tarsus, prolateral view (arrows indicate tarsal spurs).

**Figure 6:**
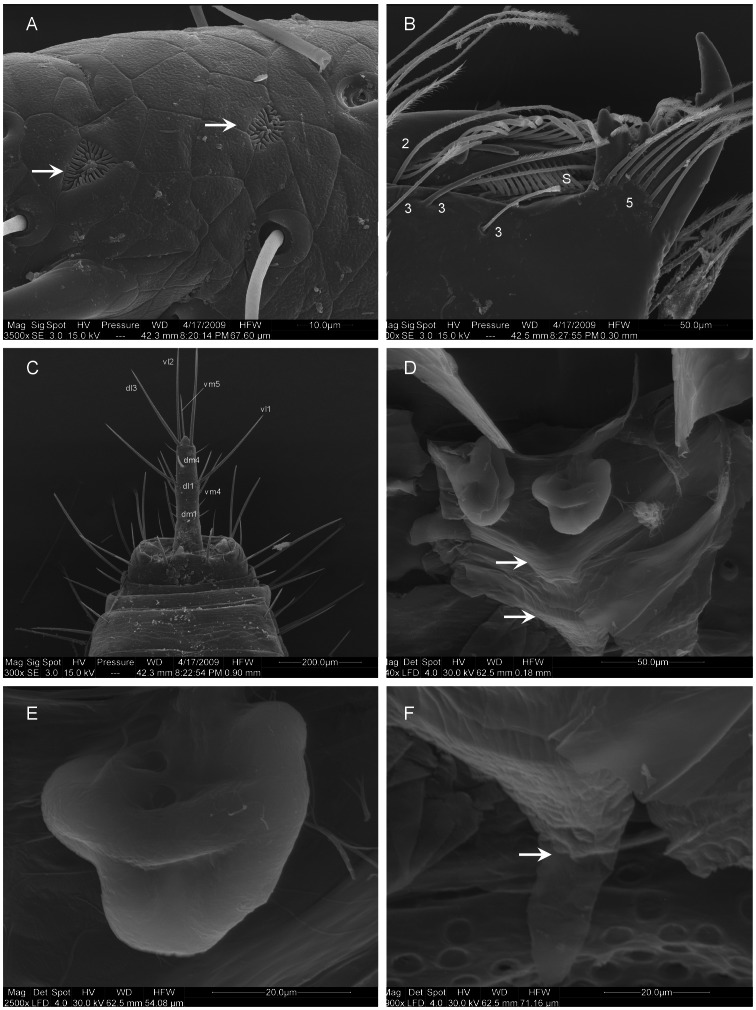
*Rowlandius potiguar* sp.nov. (A) Male (UFMG 3901), pedipalp, tarsus, prolateral view (arrows indicate uropygid pores), (B) chelicerae, apex, prolateral view (S serrula; numbers indicate setae types, according to [28]). (C) Female (UFMG 3902), flagellum and posterior tip of abdomen, dorsal view; (D) internal genitalia, dorsal view (arrows indicate chitinized arch); (E) lateral lobe of spermathecae, bulb; (F) gonopod (arrow), dorsal view.

**Figure 7:**
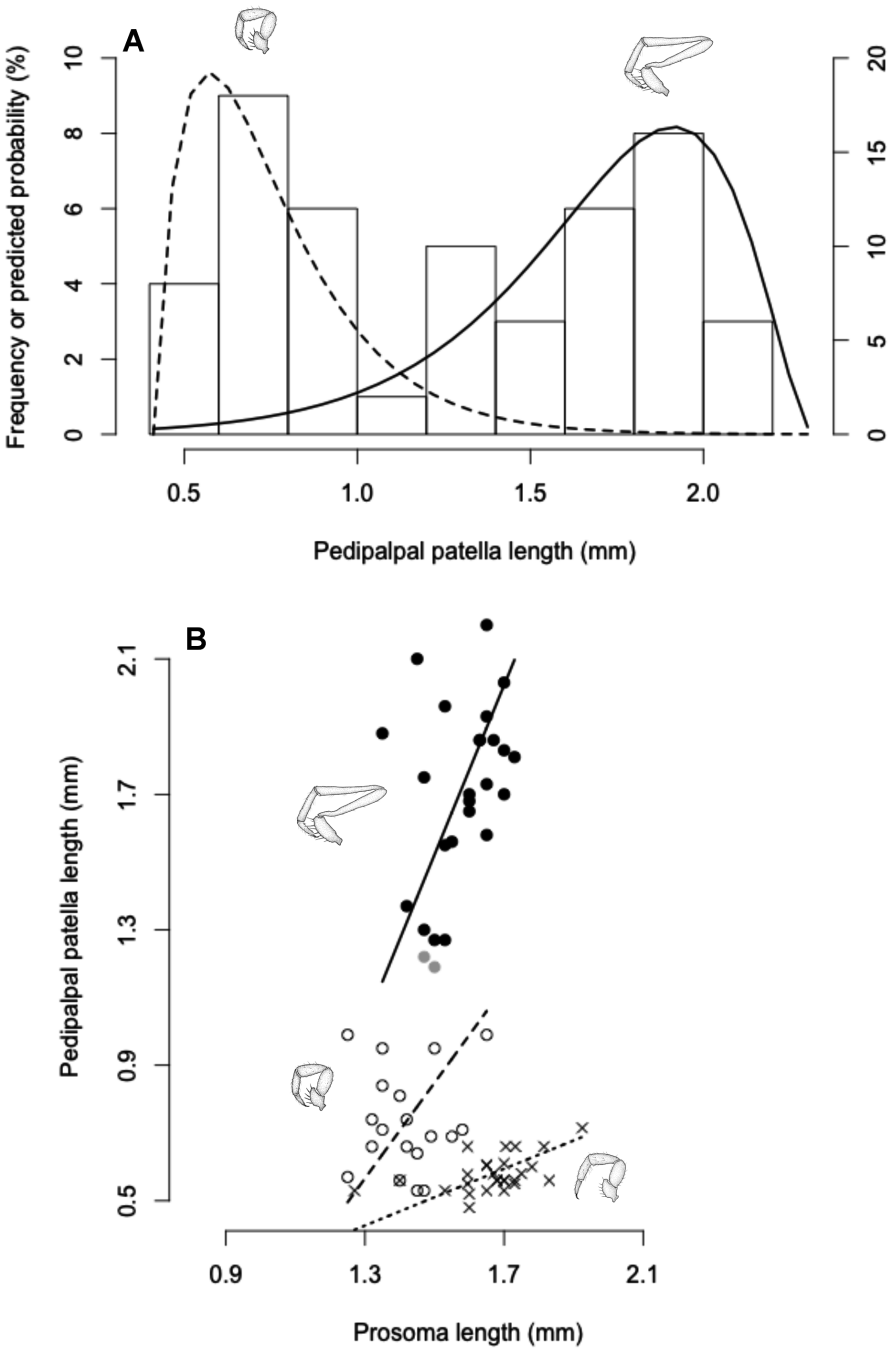
The allometry of pedipalp length in *Rowlandius potiguar* sp.nov., indicating male dimorphism. (A) Histogram of male pedipalpal patella length overlaid by the two ‘facing gamma distributions’ (θ = 0.163, λ = 1.91, lower bound = 0.48 mm for homeomorphic males; θ = 0.201, λ = 2.88, upper bound = 2.25 mm for heteromorphic males) estimated by a finite mixture model (as proposed by Rowland & Qualls [33]). The bars represent the frequency (scale on the left) of males in each pedipalpal patella length bin, and the curves represent the distributions of predicted probabilities (scale on the right, in %) of each pedipalpal patella length for both male morphs, as estimated by the model. (B) The relationship between the length of pedipalpal patella and the length of prosoma. Filled black dots and black line indicate males with a probability of being heteromorphic higher than 95%, empty dots and dashed line indicate males with a probability of being homeomorphic higher than 95%, filled gray dots indicate males with probabilities lower than 95% of being either morph, and crosses and dotted line indicate females. Axes are isometric to show male morphs in the most objective fashion.

urn:lsid:zoobank.org:act:93A5F371-8D0E-4FA4-9E4C`C9B8F08503F.

(Figures 1, 2B–D, 4A–G, 5A–D, 6A–F, 7A–B).

#### Type material

Holotype. Male from BRAZIL: *Rio Grande do Norte*: Felipe Guerra (Gruta da Carrapateira, 5°33′38′′S 37°39′50′′W), 28.VII.2009, M.P.A. Oliveira coll. (UFMG 3897). Paratypes. Female from BRAZIL: *Rio Grande do Norte*: Felipe Guerra (Gruta do Geílson, 5°35′53′′S 37°41′18′′W), 16.VI.2008, M.P.A. Oliveira coll. (UFMG 3898); male and female, same data as the holotype (UFMG 3899); male and female, ditto (IBSP 45); male and female from BRAZIL: *Rio Grande do Norte*: Baraúna (Gruta dos Cipós, 5°2′40′′ 37°34′35′′W); D.M. Bento coll. (ISLA 1837).

### Additional Material Examined

BRAZIL: *Rio Grande do Norte*: Baraúna (Gruta do Britador, 5°1′26′′S 37°29′50′′W); 11/VI/2010; D.M. Bento coll.; 2 males 2 females 2 juv (ISLA 1843); (Gruta do Pinga, 5°3′8′′ 37°32′23′′W); 28/I/2010; D.M. Bento coll.; 1 male 1 female 1 juv. (ISLA 1835); (Gruta dos Cipós, 5°2′40′′ 37°34′35′′W); D.M. Bento coll. 1 male 1 female 2 juv. (ISLA 1850); (Gruta Furna Feia; 5°2′12′′S 37°33′37′′W); 29/I/2010; D.M. Bento coll.; 2 males 2 females 1 juv. (ISLA 1834); (Gruta do Lago; 5°2′11′′ 37°34′15′′W; 26/I/2010; D.M. Bento coll.; 1 male 5 juv. (ISLA 1846); ditto; 30/VII/2010; D.M. Bento coll.; 1 male 1 female 2 juv. (ISLA 1840); (Gruta Esquecida, 5°2′20′′S 37°33′41′′W); D.M. Bento coll.; 1 male 2 females 1 juv. (ISLA 1826); same details, 12/VI/2010; D.M. Bento coll.; 1 male 1 female 3 juv. (ISLA 1838); Felipe Guerra (Gruta da Carrapateira, 5°33′38′′S 37°39′50′′W), 28.VII.2009, 4 males 2 females (UFMG 3900); (Gruta do Arapuá, 5°31′48′′S 37°36′58′′W); 3/VIII/2010; D.M. Bento coll.; 1 female (ISLA 1828); ditto, 7/I/2010; D.M. Bento coll.; 3 males 4 females (ISLA 1831); (Gruta Beira Rio, 5°33′7′′ 37°37′43′′W); D.M. Bento coll.; 2 males 2 females 2 juv. (ISLA 1822); (Gruta do Crotes, 5°33′39′′S 37°39′32′′W); 19/I/2010; D.M. Bento coll.; 3 males 3 females 1 juv. (ISLA 1827); ditto; 4/VI/2010; 1 female 2 juv. (ISLA 1832); ditto; no date, 1 female (ISLA 1847); (Gruta do Geílson, 5°35’53”S 37°41’18”W), 16.VI.2008, R.L. Ferreira coll., 6 males 2 females 7juv. (UFMG 3901); (Gruta do Buraco Redondo, 5°34′43′′S 37°39′5′′W); D.M. Bento coll.; 1 male (ISLA 1825); (Gruta Lapa 1, 5°33′42′′S 37°41′42′′W); D.M. Bento coll.; 1 male (ISLA 1821); (Gruta da Rumana; 5°33′54′′S 37°39′7′′W); 10/I/2010; D.M. Bento coll.; 2 males 1 female (ISLA 1842); Governador Dix-sept Rosado (Gruta da Boniteza, 5°30′51′′S 37°33′22′′W); 2/II/2009; D.M. Bento coll.; 1 male 2 juv. (ISLA 1829); (Gruta Capoeira do João Carlos, 5°30′57′′S 37°31′42′′W); D.M. Bento coll.; 1 male 4 females (ISLA 1823); ditto; 3/6/2010; 2 females 4 juv. (ISLA 1845); (Gruta do Lajedo Grande, 5°27′42′′S 37°33′14′′W); D.M. Bento coll.; 1 females (ISLA 1820); (Gruta do Marimbondo Caboclo, 5°29′44′′S 37°32′42′′W); D.M. Bento coll.; 4 males 5 females 1 juv. (ISLA 1824); ditto; 20/VII/2010; D.M. Bento coll.; 3 males 2 females 4 juv. (ISLA 1844); Martins (Gruta Casa de Pedra, 6°4′17′′S 37°52′59′′W), VI.2008, R.L. Ferreira coll., 4 males 6 females 1juv. (UFMG 3902); Mossoró (Gruta do Trinta, 5°12′44′′S 37°15′51′′W); 10/VI/2010; D.M. Bento coll.; 1 female (ISLA 1830).

### Etymology

The specific epithet is an adjective used in Brazil to designate the natives from the state of Rio Grande do Norte.

### Diagnosis

Males of *Rowlandius potiguar* resemble those of *R. linsduarteae* Santos *et al*., 2008 in the subquadrate flagellum, but differ in its rounded sides, dorsal projections narrower in dorsal view and the setae dm4 closer to the posterior border (Fig. 4A–C, 5A). The flagellum of this species is also similar to that of *R. monticola* Armas, 2002 in general shape (fig. 3b in [41]), but differs in the wider stalk and the dorsal projections closer to each other. Females are similar to those of *R. sul* Cokendolpher & Reddell, 2000 and *R. linsduarteae* in the conical shape of the median lobes of the spermathecae (fig. 6 in [42]) and specifically to *R. linsduarteae* in the lateral lobes of the spermathecae with curved stalks and large bulbs (figure 6 in [2]). These species can be distinguished by the lateral lobes with thinner stalks and larger bulbs and by the presence of a gonopod in *R. potiguar* (Figs. 4G, 6D–F). As in several other species of *Rowlandius*, male pedipalp are variably longer than in the female (Figs. 2B, C; 4D, E; 7A, B – see discussion below). However, unlike some species, such as *R. gladiger* (Dumitresco, 1977), *R. biconourus* (Rowland & Reddell, 1979) *R. longipalpus* (Rowland & Reddell, 1979) and *R. falcifemur* Teruel, 2003 (figs. 51, 55, 57 in [14], fig. 7 in [15]), neither the trochanter nor the femur has anterior projections (Fig. 4D, E).

### Description

Male (holotype). Prosoma, pedipalp and leg I reddish-brown. Opisthosoma, chelicerae and legs II–IV greenish brown (Fig. 2B, C). Anterior process with 1+1 setae. Propeltidium with an anterior and a posterior pair of setae. Eyespot inconspicuous, almost circular. Opisthosomal tergites I–IX each with a dorsal pair of setae, X–XI with a pair of lateral setae and a transversal row of five setae, XII with a dorsal and two lateral pairs of setae and a ventral row of four. Segments XI–XII telescoped. Posterodorsal abdominal process short, subtriangular (Fig. 4A). Fixed digit of chelicerae with seven teeth, the ventral the longest. Chelicerae setation: 1: 3; 2: 5; 3: 3; 4: 2; 5: 7; 6: 1. Setae 1 with spicules, setae 3 pilose in the apex. Movable digit with serrula and a row of pilose bristles, guard tooth conspicuous. Chelicerae with five apical teeth, the ventral one the longest (Fig. 6B). Prolateral spur of pedipalp conical, attenuated apically (Fig. 5C). Tarsal spurs of pedipalp as long as half the length of tarsal claw (Fig. 5D). Dorsal and lateral setae of pedipalp tarsus smooth, ventral setae pilose (Fig. 5D). Uropygid pores present in tarsus of leg I and tarsus of pedipalp (Fig. 6A) and in the flagellum (Fig. 5B). Leg tarsal segments proportion 2:1:2. Femur IV robust, anterior margin produced dorsally, approximately two times longer than wide. Total length 3.58. Propeltidium 1.09 long, 0.64 wide. Prosoma 1.55 long, opisthosoma 2.03. Length of pepipalpus segments: trochanter 0.53/femur 1.23/patella 1.17/tibia 0.66/tarsus 0.41/total 4.00. Length of leg segments: **I** trochanter 0.23/femur 1.07/patella 1.37/tibia 1.02/basitarsus 0.31/telotarsus 0.48/total 4.48. **II** 0.11/0.74/0.43/0.43/0.43/0.34/2.48. **III** 0.13/0.69/0.33/0.23/0.41/0.33/2.12. **IV** 0.15/1.07/0.43/0.69/0.64/0.38/3.36. Setation of flagellum: dm1, dm4; dl1, dl3; vm1, vm2, vm4, vm5; vl1, vl2 (Fig. 4A–C). One pair of dorso-lateral microsetae adjacent to dl1, six lateral microsetae between dl1 and dl3 (Fig. 5A, B, only five visible in the figure). Setae with spicules, microsetae smooth (Fig. 5B).

Female (paratype, UFMG 3898). As in male (Fig. 2D), except by the following. Flagellum with four segments, setation as in male except by three pairs of lateral microsetae: one pair in segment II (adjacent to dm1); one in III and one in IV (between dm4 and dl3) (Fig. 6C). Setae with spicules, microsetae smooth. Total length 4.17. Propeltidium 1.14 long, 0.64 wide. Prosoma 1.7 long, opisthosoma 2.47. Length of pedipalp segments: trochanter 0.2/femur 0.5/patella 0.58/tibia 0.48/tarsus 0.25/total 2.01. Length of leg segments: I trochanter 0.2/femur 0.97/patella 1.25/tibia 0.94/basitarsus 0.28/telotarsus 0.46/total 4.1. II 0.09/0.76/0.41/0.41/0.36/0.25/2.28. III 0.13/0.61/0.33/0.33/0.38/0.28/2.06. IV 0.15/1.04/0.46/0.71/0.53/0.33/3.22. Chitinized arch of internal genitalia closed and v-shaped (Fig. 6D), lateral lobes of spermathecae globular (Fig. 6E), gonopod membranous, with a pair of basal pores (Fig. 6F). Spermathecae without gland duct openings (Fig. 4G).

### Remarks

A peculiar lobed aperture was described from the appendages of Schizomida and Thelyphonida by Santos & Pinto-da-Rocha [27] and was proposed as a potential synapomorphy of Uropygi. This peculiar aperture has been reported from the tarsus of leg I and the flagellum, in both males and females of three schizomid and two thelyphonid genera. In the current study, we also report this structure in the male pedipalp of *R. potiguar* (Fig. 6A). Although Santos & Pinto-da-Rocha [27] suggest this aperture could be a glandular opening, its function remains unclear. Thus, we here propose to refer to this structure simply as the uropygid pore.

### Variation

Length of prosoma: male (N = 45) 1.25–1.73, female (N = 17) 1.27–1.83. Length of pedipalpal patella: male 0.53–2.2 (see Fig. 7), female 0.52–0.6.

### Natural History

This species is distributed in 20 caves along the Apodi limestone group, in the western part of the state of Rio Grande do Norte. The area is located in the caatinga, a seasonally-dry forest formation [16]. This limestone group is composed of huge outcrops, located in various municipalities, including Jandaíra, Felipe Guerra, Apodi, Martins and Baraúnas. Populations of *R. potiguar* were found in many caves (Fig. 1), although the population sizes were variable, possibly due to different conditions in each cave. The species is apparently associated with moist caves, since no specimens have been observed in completely dry caves. In the dry Casa de Pedra cave, the southernmost record of the species, the single specimen observed was in the only wet area of the cave, in sediments humidified by dripping water. Field observations suggest that this species occurs in large populations, with hundreds of individuals, particularly close to bat guano or seed deposits transported by bats. These organic piles harbor several scavenger invertebrates, like springtails (Collembola), small flies (Diptera) and booklice (Psocoptera); which could serve as prey for schizomids.

### Distribution

Known from 20 caves in the northeastern Brazilian state of Rio Grande do Norte (Fig. 1).

### Male Dimorphism

Pedipalp length in *R. potiguar* males is remarkably variable, presenting 20.11 times more variance than prosoma length (variances standardized by the means of the traits), and its distribution is clearly bimodal (Figure 7A). Meanwhile, the same trait in conspecific females presents a unimodal distribution, with only 0.50 of the variance presented by prosoma length (variances standardized by the means of the traits). Moreover, a mixture of two ‘facing gamma distributions’ (θ = 0.163, λ = 1.91, lower bound = 0.48 mm for homeomorphic males; θ = 0.201, λ = 2.88, upper bound = 2.25 mm for heteromorphic males; Figure 7A) fits the distribution of male pedipalp length very well. This model estimates that at least 95% of males with pedipalpal patella shorter than 1.02 mm represent the homeomorphic morph (empty circles, Figure 7B) and at least 95% of the males with pedipalpal patella longer than 1.23 mm represent the heteromorphic morph (full black circles, Figure 7B).

The only known cases of elongated male-dimorphic traits in arachnids were described for the order Opiliones [22], [23], where the elongation of the second or fourth pair of legs is bimodal and extremely variable among males. In these cases, the male dimorphic traits are sexually selected weapons used in male-male fights. As far as we know, there is no evidence of male-male fights in Schizomida, and the only studies that describe the courtship and copulation in the order [43], [44] do not indicate any participation of the male pedipalp in mating. However, there is evidence that males of the schizomid *Hubbardia pentapeltis* use their pedipalps during courtship. In this species, the male stretches out his pedipalps, picks up small twigs, and manipulates them in various ways while the female seemingly examines the male’s behavior, repeatedly touching his anterior body parts with her first legs (JM Rowland, personal communication). In conclusion, it is also possible that the elongation of pedipalps in *R. potiguar* evolved in the context of courtship.

The fact that the static allometry of pedipalps is significantly steeper in males than in females (Figure 7, Table 1) cannot be readily taken as evidence that such pedipalps are under sexual selection [45], as it has been interpreted in the past for other taxa [46]. However, it does indicate that pedipalp length is under a different selective regime in males than in females. We urge for studies on the sexual behavior of *R. potiguar* and other schizomids, which would be promising approaches to shedding light on the evolutionary causes of pedipalp elongation and male dimorphism in the group.

**Table 1:** Coefficients (and their respective 95% confidence intervals) from the simple linear models fitted to the allometric relationship between pedipalp length and prosoma length for each male morph and for females.

	Intercept			Slope		
	Estimate	Lower 92.5% CI	Upper 97.5% CI	Estimate	Lower 97.5% CI	Upper 97.5% CI
Heteromorphic males	−2.229	−4.204	−0.911	2.500	1.668	3.750
Homeomorphic males	−1.268	−2.537	−0.491	1.411	0.864	2.305
Females	−0.117	−0.410	0.090	0.418	0.294	0.595

Models were fitted through standard major axis regression. Note that the slopes estimated for heteromorphic and homeomorphic males are significantly higher than that estimated for females.

In conclusion, an objective and robust discrimination of male morphs might be crucial for in depth behavioral and ecological studies of particular male dimorphic species, but it does not seem to be so important for taxonomic descriptions. Nevertheless, it is still essential that taxonomists realize the existence of male dimorphism, and hence avoid describing male morphs as different species. This does not seem to be a problem in schizomid systematics, since species distinction is based mainly on male flagellum. Yet, it is relevant to report variation in male pedipalp length within species and, whenever a reasonably big sample is available, discriminate morphs properly. The detection of male dimorphism by taxonomists can in itself stimulate behavioral ecology studies on these animals, which could elucidate their mating systems and unveil the alternative mating tactics of males that are potentially behind the evolution of male dimorphism in Schizomida.
